# The Gut–Pancreas Axis in Type 1 Diabetes: Emerging Insights into Microbiota and Immune Interactions

**DOI:** 10.3390/ijms27114789

**Published:** 2026-05-26

**Authors:** Rahul Mittal, Priyanka Sinha, Jhanvi Doshi, Rebecca Goldmann, Mannat Mittal, Naisha Chaudhary, Vibha Ravindra, Khemraj Hirani

**Affiliations:** 1 Diabetes Research Institute, University of Miami Miller School of Medicine, Miami, FL 33136, USA; priyankasinha.ras@gmail.com (P.S.); jxd4468@miami.edu (J.D.); 0528001@dadeschools.net (M.M.); naishac2010@gmail.com (N.C.);; 2 Division of Endocrinology, Diabetes, and Metabolism, Department of Medicine, University of Miami Miller School of Medicine, Miami, FL 33136, USA

**Keywords:** type 1 diabetes, gut microbiota, gut–pancreas axis, β-cell autoimmunity, microbial metabolites, host–microbial interactions, immune regulation

## Abstract

The gut microbiota is increasingly recognized as an important factor in the pathogenesis of type 1 diabetes (T1D), although its exact role in disease initiation and progression remains uncertain. Earlier interpretations considered alterations in intestinal microbial composition as secondary effects of immune dysregulation or metabolic disturbance. Recent longitudinal studies, however, suggest that specific microbial changes occur before the onset of islet autoimmunity, indicating a potential contributory role in the early phases of disease development. In this narrative review article, the gut–pancreas axis (GPA) is described as a dynamic and reciprocal system in which microbial, metabolic, and immune processes influence each other to shape β-cell outcomes. Evidence from human cohorts and experimental models links early life reductions in microbial diversity, impaired intestinal barrier function, and decreased production of short-chain fatty acids (SCFAs) to altered immune activation and β-cell damage. Microbiota transferred from individuals at risk for T1D has been shown to accelerate disease in animal models, supporting a possible causal relationship. Although experimental models support mechanistic links between microbiota alterations and autoimmune diabetes, current human evidence remains largely associative. Together, these findings suggest that microbial and immune networks interact in a feedback manner that can sustain immune tolerance or promote autoimmunity depending on environmental and host factors. Understanding T1D as a state of disrupted microbial and immune integration provides a basis for restoring gut–pancreas communication and preserving β-cell integrity.

## 1. Introduction

Type 1 diabetes (T1D) is an autoimmune disease characterized by the destruction of pancreatic β-cells, leading to absolute insulin deficiency and chronic hyperglycemia [1,2,3]. Although genetic predisposition remains a major determinant of risk, most notably the human leukocyte antigen (HLA) class II alleles DR3 DQ2 and DR4 DQ8, heritability alone cannot account for the rising global incidence of the disease or its geographic variability [4]. Importantly, T1D is now widely recognized as a heterogeneous condition encompassing distinct age-related endotypes that differ in immunological signature, β-cell pathology, and rate of progression [5]. This heterogeneity has direct implications for any unifying pathogenic model as the relative contribution of microbial, immune, viral, and metabolic factors is unlikely to be uniform across endotypes. Consequently, disease initiation and progression are more accurately conceptualized as the outcome of context-dependent interactions between genetic susceptibility and environmental exposures, which collectively modulate disease penetrance and trajectory. Among environmental factors, gut microbiota has emerged as a central regulator of immune development, influencing tolerance induction, antigen presentation, and inflammatory responses [6,7,8,9,10,11]. These processes are defined here as immune regulation, encompassing cellular and molecular mechanisms that constrain excessive immune activation while preserving effective host defense. Within this framework, the intestinal microbiota represents a metabolically active ecosystem that interfaces with epithelial and immune cells through microbial metabolites and pattern recognition pathways, thereby shaping both innate and adaptive immunity [12,13].

Historically, alterations in gut microbial composition, or dysbiosis, observed in individuals with T1D were interpreted as secondary phenomena resulting from metabolic instability, dietary modification, or systemic inflammation following disease onset [6]. In this review we use the term dysbiosis specifically to denote a reproducible, disease-associated departure from age- and population-matched microbial reference communities, characterized by quantifiable changes in diversity, the abundance of defined commensal taxa, or microbial functional gene content. Consistent with the earlier framework, metabolic disturbances such as hyperglycemia and inflammation were proposed to reshape the intestinal environment and drive microbial imbalance [7]. However, this unidirectional model has been increasingly challenged by data from large-scale prospective birth cohorts that have monitored microbial development in genetically at-risk children prior to disease onset [8]. Longitudinal analyses from studies such as the Environmental Determinants of Diabetes in the Young (TEDDY), the Type 1 Diabetes Prediction and Prevention (DIPP), and BABYDIET demonstrate that alterations in microbial composition and function precede the development of islet autoimmunity by months to years [9,10,11]. These early perturbations include reduced microbial diversity, depletion of short-chain fatty acid-producing commensals such as *Faecalibacterium prausnitzii* and *Roseburia*, and enrichment of proinflammatory taxa such as *Bacteroides dorei*.

Functional metagenomic and metabolomic data further reveal that these compositional changes are accompanied by loss of pathways related to butyrate and tryptophan metabolism, both of which play central roles in maintaining epithelial integrity and regulatory immune tone [12,13,14,15]. This state, referred to here as immune homeostasis, is defined as the dynamic equilibrium between pro-inflammatory and tolerogenic immune responses across mucosal and systemic compartments. Within this context, the temporal precedence of these alterations supports a model in which microbial dysfunction contributes to the initiation, rather than merely the consequence, of immune dysregulation [16,17,18]. Experimental evidence from animal models provides direct support for the causal role of gut microbiota in autoimmune diabetes. Early studies in non-obese diabetic (NOD) mice demonstrated that host–microbial interactions, particularly through innate immune pathways, critically regulate disease susceptibility, establishing a mechanistic link between microbial exposure and autoimmune pathogenesis [19,20]. Subsequent studies showed that environmentally induced perturbations of microbiota, including antibiotic-mediated alterations, significantly modify diabetes incidence and progression [21,22]. Transfer of microbiota from at-risk or diabetic donors accelerates insulitis and disease onset in recipient mice, whereas restoration of defined microbial communities or supplementation with microbial metabolites promotes immune regulation and reduces disease incidence [12,23,24,25,26]. It has been further demonstrated in experimental systems that microbiota-dependent pathways involving microbial metabolites, antigenic mimicry, and translocation of microbial components to pancreatic lymphoid tissues can influence β-cell-specific immune responses [15,27,28]. Genome-wide and integrative multi-omic analyses, including metagenomic sequencing from the TEDDY cohort and metagenome-assembled genome studies, further support these observations [29,30,31]. Collectively, findings suggest that functional alterations in microbial gene content and metabolic pathways precede islet autoimmunity and interact with host and environmental factors to shape disease risk.

**Figure 1:**
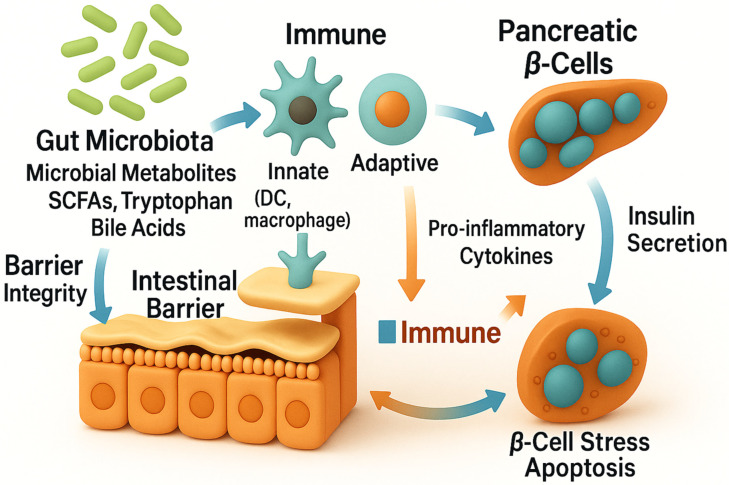
Gut Pancreas Axis in Metabolic and Immune Homeostasis. A schematic representation of the bidirectional communication between the gut microbiota, intestinal barrier, immune system, and pancreatic β-cells. Gut microbial metabolites such as short-chain fatty acids (SCFAs), tryptophan derivatives, and bile acids support intestinal barrier integrity and modulate immune responses. Immune-mediated signaling and proinflammatory cytokines influence β-cell function, leading to either insulin secretion or β-cell stress and apoptosis. This dynamic interplay highlights how gut immune pancreatic crosstalk contributes to metabolic homeostasis and the pathogenesis of Type 1 diabetes (T1D). Created in BioRender. Mittal, R. (2026) https://BioRender.com/y8udoyc (accessed on 10 April 2026).

The objective of this narrative review article is to integrate longitudinal and experimental data to redefine the gut–pancreas axis (GPA) as a bidirectional ecological and immunological system in T1D (Figure 1). The review further aims to examine the relationships between microbial perturbations, barrier dysfunction, and β-cell autoimmunity, and to highlight testable hypotheses for future investigations. In this context, support for mechanistic relationships is derived primarily from experimental models, whereas corresponding findings in humans remain predominantly associative. Therefore, the pathways discussed throughout the manuscript should be interpreted cautiously and should not be regarded as established drivers of disease in humans. Taken together, this framework highlights the need for integrative approaches spanning microbial ecology, immunology, and metabolism to advance conceptual understanding of GPA and inform therapeutic development.

## 2. Conceptual Positioning of the Gut–Pancreas Axis

The GPA is conceptualized as an organ-resolved network that integrates microbial ecology, mucosal immunology, and pancreatic endocrine biology within a unified framework [32,33,34]. This model extends beyond generalized host–microbiome paradigms by explicitly positioning the pancreatic islet as both a target and an active participant in systemic host–microbial crosstalk [35]. In contrast to traditional frameworks that emphasize signaling from the gut microbiota to peripheral immune compartments, the GPA incorporates reciprocal loops in which pancreatic-derived factors may influence intestinal physiology and microbial composition [36,37].

At a mechanistic level, the GPA is defined by the convergence of microbial metabolites, immune cell trafficking, cytokine networks, and neuroendocrine pathways [38,39]. These signals connect the intestinal lumen, mucosal immune system, and pancreatic microenvironment, positioning β-cell function within a broader ecological and immunological network [40]. The pancreas, in this context, is embedded within a broader system-level network in which perturbations in microbial community structure or function can propagate through metabolic and immunological pathways to influence β-cell fate.

The GPA also introduces a higher degree of organ specificity compared to broader host–microbiome–metabolic frameworks [39]. While existing models capture systemic interactions between microbiota and host physiology, they often lack resolution with respect to how these interactions are integrated at the level of individual organs and disease-relevant cellular targets. By centering the pancreatic islet within this network, the GPA provides a mechanistic scaffold for understanding how microbial and immune perturbations converge on β-cell–specific processes, including antigen presentation, immune recognition, and cellular stress responses. This organ-focused perspective is particularly relevant in the context of T1D, where disease pathogenesis is defined by highly specific immune-mediated destruction of β-cells despite systemic immune activation.

Comparison with the gut–liver axis further contextualizes the GPA within established models of inter-organ communication. The gut–liver axis is supported by well-characterized anatomical and physiological connections, including direct portal venous flow, enterohepatic circulation of bile acids, and continuous exposure of the liver to gut-derived metabolites and microbial products [41,42,43,44,45,46]. These features enable relatively direct and quantifiable interactions, facilitating mechanistic dissection and translational application across a range of liver diseases [47,48,49]. In contrast, communication within the GPA is mediated through more distributed pathways, including systemic circulation of microbial metabolites, lymphatic trafficking of immune cells, and antigen presentation within pancreatic draining lymph nodes [50,51,52,53,54,55,56,57]. Although these routes are less anatomically direct, they are supported by convergent experimental and clinical data suggesting functional connectivity between the gut and pancreatic immune compartments.

Importantly, the absence of a singular anatomical conduit does not preclude the existence of a functionally coherent axis. Rather, it suggests that the GPA operates as a networked system in which multiple parallel pathways collectively mediate inter-organ communication [19,58]. This distributed architecture may confer both complexity and adaptability, allowing for integration of diverse environmental, microbial, and host-derived signals. However, it also presents challenges for mechanistic resolution, as individual pathways may exert context-dependent effects that are difficult to isolate in human studies.

From a translational perspective, the GPA provides a framework for integrating longitudinal microbiome data with mechanistic insights from experimental models, while recognizing that human evidence remains primarily associative.

Collectively, the GPA represents an integrative, system-level model that emphasizes organ specificity and the convergence of microbial, metabolic, and immune pathways. This framework advances current understanding by providing a structured approach to investigating how distal ecological interactions may influence pancreatic autoimmunity and endocrine function.

## 3. Interpretation of Causality and Strength of Evidence

The relationship between alterations in the gut microbiota and the development of T1D requires careful interpretation with respect to causality. A central limitation of the existing literature, repeatedly emphasized in recent critical appraisals [10,59], is that direct causal evidence linking gut microbiota to T1D in humans remains scarce. Most cohort studies are observational, and although longitudinal cohorts show that microbial changes may precede the development of islet autoimmunity, these data do not establish direct causation. Experimental studies, particularly in NOD mouse models and humanized gnotobiotic systems, suggest that targeted manipulation of microbial communities and their metabolites can influence disease onset and progression. However, mechanistic insights from model systems should be interpreted as biologically plausible pathways that require validation in human contexts.

A clear distinction between observational human data and mechanistic findings derived from experimental systems is therefore essential. Experimental models permit controlled perturbation of microbial and immune pathways, enabling causal inference under defined conditions, whereas human studies are influenced by substantial biological and environmental variability that limits direct attribution of cause and effect. Interpretation of microbiome–disease associations must therefore account for well-defined confounding factors that influence both microbial composition and disease risk.

Diet. Both habitual diet and the timing of gluten and complementary food introduction influence microbial composition and islet autoimmunity. Pilot dietary interventions in BABYDIET found that the timing of gluten introduction did not, in their cohort, significantly alter islet autoimmunity risk [60,61]; in contrast, recent DIPP analyses suggest that lower dietary fibre intake is associated with greater risk of progression to islet autoimmunity [62]. Diet therefore acts as a partial confounder both for microbial composition and for autoimmune outcomes, and microbial signatures cannot be interpreted independently of dietary exposure history.

Antibiotic exposure. Early-life antibiotic exposure has reproducible effects on the developing microbiome, with separate observational data linking it to increased T1D risk in children [63]. In NOD mice, antibiotic-induced microbiota perturbation is sufficient to accelerate diabetes [22], demonstrating biological plausibility, but in humans the magnitude of effect remains modest and may be confounded by the underlying infections for which antibiotics are prescribed.

Mode of delivery and breastfeeding. Caesarean delivery has been associated with altered neonatal microbial colonisation and a small but reproducible increase in T1D risk [64], while breastfeeding shapes the developing microbiota and modulates infant mucosal immunity, including via human milk IgA-mediated control of Th17-inducing commensals [65]. These early-life exposures are systematically distributed by socioeconomic and geographic factors and therefore confound any straightforward microbiota–disease association.

Geographic variation and the hygiene gradient. The 5- to 6-fold gradient in T1D incidence between Finland and neighbouring Russian Karelia, despite genetic similarity, has been linked to differences in early-life microbial exposure and to a more immunogenic LPS structure in Finnish infant *Bacteroides* communities [66,67]. Such observations indicate that geographic variation in microbiota cannot be reduced to a single quantitative axis of “diversity” and that ecological context modifies the host immunological consequences of any given microbial community.

Host genetic background. HLA risk alleles themselves shape gut microbial composition. In Mexican schoolchildren stratified by HLA-DQ2/DQ8, high-risk haplotypes were associated with reduced phylogenetic diversity and altered *Akkermansia* and *Parabacteroides* abundance [68], and adults with high-risk DR3/DR4/DR9 combinations show distinct microbial and serum metabolite signatures relative to lower-risk genotypes [69]. A multicentre analysis demonstrated that genetic risk for autoimmunity is associated with discrete microbial changes independently of disease status [70]. Microbiota-based risk inference must therefore be performed within HLA strata to avoid confounding by genotype-driven community variation.

Disease heterogeneity. Even within HLA-stratified cohorts, T1D is not a single disease. Endotype frameworks distinguish age <7 vs. ≥13 endotypes by histological and immunological features [71] and recent systematic frameworks recognize at least six provisional endotypes defined by age, autoantibody pattern, β-cell loss kinetics, and exposome [5,72]. The contribution of microbiota-mediated mechanisms is unlikely to be uniform across these endotypes, a point that should be reflected in the design of any future biomarker or interventional study.

Taken together, these confounders do not negate the evidence for a microbial contribution to T1D, but they impose strict limits on causal inference from observational data. Failure to account for diet, antibiotic exposure, mode of delivery, breastfeeding, geographic and environmental variation, host genetic background, and disease heterogeneity may lead to overestimation of the strength or specificity of microbiota-related associations. The combination of reproducible temporal precedence of microbial change, biological plausibility from defined mechanisms in NOD mice and humanized gnotobiotic systems, and early signals from interventional fecal microbiota transplantation (FMT) and probiotic studies provide convergent but still incomplete evidence. A rigorous assessment of causality will require integrative approaches that combine longitudinal human cohorts with mechanistic and interventional designs, enabling more precise delineation of the pathways linking microbial perturbations to β-cell autoimmunity.

## 4. Data from Longitudinal Cohorts

Extensive longitudinal investigations have provided robust evidence that alterations in gut microbial composition and function preceded the onset of autoimmunity in individuals who later develop T1D [73,74,75]. The TEDDY study, one of the most comprehensive multicenter prospective cohorts, followed thousands of genetically predisposed infants from birth and performed dense temporal sampling of their intestinal microbiota [9,16,29,30,76]. Analyses revealed that children who eventually developed islet autoantibodies displayed a marked reduction in overall microbial diversity months before seroconversion, accompanied by depletion of beneficial commensal genera such as *Bifidobacterium* and *Lactobacillus* [7,74]. These taxa are known to contribute to epithelial barrier maintenance, short-chain fatty acid production, and immune tolerance. In parallel, there was a relative enrichment of *Bacteroides dorei* and other proinflammatory species that can promote mucosal immune activation through production of lipopolysaccharide variants with heightened toll-like receptor signaling potential.

Comparable trends were identified in other independent prospective studies, including the DIPP and BABYDIET cohorts [29,73,74,77,78]. These investigations demonstrated that children who developed autoimmunity exhibited delayed microbiota maturation, characterized by a persistence of early infant-type microbial configurations and a failure to transition toward a more complex, adult-like community structure. In these cohorts, reductions in butyrate-producing organisms such as *Faecalibacterium prausnitzii*, *Roseburia intestinalis*, and *Eubacterium rectale* were particularly notable [73,74]. The loss of these organisms corresponded with diminished abundance of microbial genes involved in butyrate biosynthesis pathways, an observation that has functional implications for intestinal barrier integrity and immune homeostasis [79]. Butyrate and related metabolites serve as major energy sources for colonocytes and have well-established anti-inflammatory effects, mediated through histone deacetylase inhibition and regulatory T cell induction [80,81,82]. Their depletion therefore represents a mechanistic link between microbial dysbiosis and systemic immune dysregulation.

Metabolomic analyses in infants at risk have further identified disruptions in amino acid and lipid metabolic pathways that are consistent with microbial functional impairment [83,84,85]. In particular, reduced tryptophan catabolism into indole derivatives and kynurenine pathway intermediates have been observed prior to seroconversion [86,87,88]. These metabolites are important activators of the aryl hydrocarbon receptor (AHR), which regulates interleukin-22 (IL-22) production and mucosal tissue repair [89,90,91]. Their deficiency may therefore compromise epithelial integrity and enhance antigen translocation across the gut barrier, facilitating the activation of autoreactive lymphocytes in pancreatic draining lymph nodes [29,79,92].

Experimental evidence from animal models has strengthened the argument for causality [93]. Studies using germ-free and humanized non obese diabetic (NOD) mice have shown that transplantation of fecal microbiota from individuals at risk for T1D or from diabetic donors accelerates insulitis and disease onset in recipient animals [22,26,94]. Conversely, colonization with defined microbial consortia enriched in butyrate- or propionate-producing strains restores mucosal immune balance, enhances Foxp3-positive regulatory T cell populations, and delays or prevents diabetes development [12,15,24]. These findings demonstrate that microbial communities can directly influence the trajectory of autoimmune pathology through modulation of host immune networks and intestinal physiology.

Taken together, data from longitudinal human cohorts and mechanistic animal studies converge on the conclusion that specific compositional and metabolic features of the gut microbiota are altered well before the emergence of islet autoimmunity. These early changes are unlikely to be merely passive reflections of developing disease and may contribute to immune outcomes, although causality in humans remains unproven. The temporal precedence, biological plausibility, and experimental reproducibility of these findings collectively support but do not yet prove a causal relationship between early life microbial dysregulation and the initiation of β-cell-directed immune responses.

## 5. Mechanistic Links Along the Gut–Pancreas Axis (GPA)

The intestinal barrier serves as the principal structural and immunological interface between the gut microbiota and the host organism [95,96]. This barrier is composed of a single layer of epithelial cells sealed by tight junctions, covered by a mucus layer, and supported by immune cells residing in the lamina propria. It functions not only as a physical boundary that separates the luminal microbial community from the systemic circulation, but also as a dynamic immunological sensor that interprets microbial and dietary signals. Under physiological conditions, the integrity of this barrier maintains immune tolerance through balanced interactions between commensal microorganisms and host defense mechanisms. However, alterations in the gut microbial community can compromise epithelial stability by disrupting tight junction proteins, reducing mucin production, and modifying the secretion of antimicrobial peptides, such as defensins and regenerating islet-derived proteins. These changes weaken the mucosal defense and increase intestinal permeability, a phenomenon frequently documented in both preclinical and established T1D (Table 1). In human studies, these observations are primarily associative and do not establish direct causality between microbiota alterations and T1D development.

### 5.1. Barrier Dysfunction and Microbial Dysbiosis

Recent human studies further support the contribution of intestinal barrier dysfunction and microbial dysbiosis to T1D pathogenesis by demonstrating coordinated alterations in gut microbial composition, mucus barrier integrity, and mucosal immune responses [100]. Detailed analyses of intestinal samples from individuals with T1D revealed evidence of gut barrier impairment characterized by structural alterations of the mucus layer together with significantly reduced expression of multiple mucin genes, including *MUC2*, *MUC12*, *MUC13*, *MUC15*, *MUC20*, and *MUC21*, as well as decreased expression of antimicrobial peptides such as human defensin 4 (*DEFA4*) and human defensin 5 (*DEFA5*) [100]. These epithelial changes were associated with shifts in gut microbial composition, notably a reduced relative abundance of short-chain fatty acid–producing bacterial species, including *Bifidobacterium dentium*, *Clostridium butyricum*, and *Roseburia intestinalis*, which are known to regulate mucin production and maintain intestinal immune homeostasis. Concomitantly, immune profiling of intestinal tissues demonstrated evidence of mucosal immune dysregulation, characterized by increased frequencies of pro-inflammatory effector T-cell subsets, including T helper 1 (Th1), Th17, and TNF-α–producing T cells [100]. Taken together, these findings indicate that disturbances in microbial ecology, epithelial barrier integrity, and mucosal immune regulation occur in individuals with T1D. However, these findings are correlative and do not demonstrate that such alterations are causal drivers of disease in humans.

At a mechanistic level, epithelial barrier integrity reflects coordinated regulation of tight-junction and adherens-junction complexes, mucus-layer architecture, AMP production, epithelial renewal, and cytokine-mediated signaling. Inflammatory mediators implicated in mucosal and autoimmune inflammation, including TNF-α, IL-1β, IL-6, and IFN-γ, can disrupt junctional organization, impair epithelial polarity, reduce mucus barrier function, and increase paracellular permeability. These changes may enhance exposure of mucosal immune cells to dietary or microbial antigens, thereby linking epithelial barrier disruption to local immune activation and downstream β-cell–directed autoimmune responses. Together, these coordinated structural, microbial, metabolic, and immune alterations suggest that disruption of host–microbial homeostasis at the gut interface may contribute to the initiation or amplification of autoimmune responses targeting pancreatic β-cells.

### 5.2. Microbial Translocation and Innate Immune Activation

Loss of barrier integrity allows microbial antigens, metabolites, and structural components including lipopolysaccharide, flagellin, and peptidoglycan to translocate into the subepithelial compartment and eventually into systemic circulation [101,102,103]. The presence of these microbial-derived molecules beyond the intestinal lumen provokes immune activation through engagement of pattern recognition receptors on dendritic cells and macrophages in mesenteric and pancreatic lymph nodes [19,27,101,104]. Activation of these antigen-presenting cells promotes cross presentation of pancreatic autoantigens, providing a mechanistic link between gut inflammation and the initiation of β-cell–specific immune responses [27,28,105]. Chronic exposure to these microbial components can sustain a proinflammatory milieu characterized by the production of cytokines such as IL-6, TNF-α, and IFN-γ, thereby amplifying autoreactive T cell proliferation and perpetuating β-cell destruction [106,107,108]. While such mechanisms are well supported in experimental systems, particularly in NOD mouse models, direct evidence for this sequence of events in humans remains limited.

In addition to innate immune activation, adaptive immune mechanisms may also be influenced by microbial-derived signals.

### 5.3. Molecular Mimicry and Adaptive Immune Responses

Beyond innate immune activation triggered by translocated microbial components, adaptive immune responses may also be influenced by molecular mimicry between microbial antigens and pancreatic β-cell proteins [28,97,109,110,111]. Molecular mimicry occurs when structural or sequence similarity between microbial peptides and host autoantigens results in cross-reactive immune recognition [112]. Experimental data suggests that microbial peptides can share homology with key β-cell antigens, including insulin, glutamic acid decarboxylase (GAD65), and islet antigen-2 (IA-2), thereby enabling activation of autoreactive T lymphocytes in genetically susceptible hosts [28,97]. Microbial peptides derived from gut bacteria can activate diabetogenic CD8^+^ T cells in the NOD mouse model, leading to enhanced islet infiltration and accelerated autoimmune diabetes [28]. Subsequent analyses have further identified bacterial proteins containing motifs closely resembling the insulin B-chain epitope recognized by pathogenic T-cell receptors associated with T1D, supporting the concept that microbial peptide homology may contribute to the priming of autoreactive immune responses [97]. In the context of intestinal barrier disruption and dysbiosis, increased exposure of mucosal immune cells to microbial antigens may therefore enhance the likelihood of cross-reactive antigen presentation and autoreactive T-cell activation. Together, these observations suggest that molecular mimicry represents a potential mechanism linking alterations in gut microbial composition with the initiation or amplification of β-cell–directed autoimmunity in T1D. However, direct evidence that molecular mimicry causally drives β-cell autoimmunity in humans remains limited, and this mechanism should therefore be interpreted as experimentally supported but not yet clinically established.

In parallel, microbial metabolites themselves act as key intermediates linking microbiota composition to immune regulation.

### 5.4. Immunomodulatory Effects of SCFAs

Furthermore, microbial metabolites serve as key molecular intermediates in maintaining mucosal and systemic immune balance [113,114,115,116]. Short-chain fatty acids, particularly acetate, propionate, and butyrate, are generated through bacterial fermentation of dietary fibers and exert immunomodulatory effects [117,118,119]. Experimental evidence further supports the protective role of microbial short-chain fatty acids in autoimmune diabetes. In NOD mice, dietary interventions designed to enhance colonic production of acetate and butyrate markedly reduced the incidence of diabetes and attenuated insulitis [15]. Mechanistic analyses demonstrated that butyrate promoted the differentiation and expansion of regulatory T cells, whereas acetate limited the activation and proliferation of autoreactive T cells through modulation of antigen-presenting B-cell function. The combined immunomodulatory effects of these microbial metabolites resulted in a substantial reduction in pathogenic T-cell responses and provided protection against autoimmune β-cell destruction [15]. While these results support a causal role in experimental systems, human data linking SCFA alterations to T1D are largely associative. In addition, SCFAs reinforce epithelial barrier integrity by supporting tight-junction stability and mucin synthesis [120].

Beyond these barrier-supportive effects, SCFAs also regulate immune responses through complementary epigenetic and receptor-mediated pathways [121,122]. Butyrate can inhibit histone deacetylases (HDACs), thereby altering chromatin accessibility and promoting tolerogenic immune programs, including reduced conventional dendritic-cell development and diminished T-cell priming capacity [123,124,125]. This provides a plausible mechanism by which reduced butyrate availability may impair antigen-presenting-cell tolerance and favor expansion of autoreactive immune responses relevant to β-cell autoimmunity. In parallel, SCFAs signal through free fatty acid receptors, particularly FFAR2/GPR43 and FFAR3/GPR41, which are expressed on epithelial and immune cells and regulate inflammatory cytokine programs, dendritic-cell function, and epithelial repair responses [126,127,128]. GPR43-mediated signaling can further promote dendritic-cell amphiregulin expression through a Blimp-1–dependent pathway, suggesting a receptor-mediated route by which microbial metabolites contribute to epithelial repair and mucosal immune restraint [129]. Together, HDAC-dependent epigenetic regulation and GPR41/GPR43-mediated receptor signaling provide complementary mechanisms through which loss of SCFA-producing taxa may weaken epithelial integrity, reduce regulatory immune tone, and facilitate β-cell–directed autoimmunity [12,130]. However, although these mechanisms are supported by experimental and translational models, their causal contribution to human T1D remains incompletely established and should be interpreted as biologically plausible rather than clinically proven.

### 5.5. Tryptophan Metabolism and AHR Signaling Pathway

Tryptophan metabolism represents an additional microbiota-dependent pathway linking intestinal ecology to immune regulation in T1D [14,131,132,133]. Commensal bacteria convert dietary tryptophan into indole derivatives that activate the AHR in epithelial and immune cells, thereby regulating mucosal immune homeostasis [14,134]. Experimental studies in NOD mice further support the importance of tryptophan metabolism in diabetes pathogenesis. Defects in microbial and host tryptophan metabolic pathways were shown to alter immune regulation and promote autoimmune diabetes development in NOD mice, highlighting the critical role of tryptophan-derived metabolites in maintaining immune tolerance [20]. These experimental findings support a mechanistic role for tryptophan metabolism in autoimmune diabetes models, whereas human evidence remains largely associative. Accordingly, disruption of this pathway may represent a biologically plausible contributor to autoimmune activation mediated through AHR signaling and perturbations in mucosal immune homeostasis. Genetic variation in AHR signaling further modulates susceptibility to autoimmune diabetes. Notably, polymorphisms in the *Ahr* gene have been identified across several mouse strains, including the presence of a defective Ahr allele in NOD mice that may impair normal AHR signaling pathways involved in immune regulation [135]. Such genetic variation may further amplify the effects of dysregulated microbial tryptophan metabolism and contribute to altered immune responses that may predispose to autoimmune diabetes. AHR activation induces interleukin-22 production, which contributes to epithelial barrier integrity and regulation of mucosal immune responses [136,137,138,139]. Impairment of this axis may enhance microbial antigen exposure and facilitate activation of autoreactive immune pathways relevant to T1D [134,140,141]. However, the causal relevance of AHR-dependent tryptophan signaling in human T1D remains to be clarified.

Beyond tryptophan-derived metabolites, microbial modification of bile acids represents another pathway through which the gut microbiota may shape immune regulation.

### 5.6. Bile Acid Metabolism and Immune Modulation

Beyond tryptophan metabolism, additional microbiota-derived metabolites also participate in regulating immune responses along the gut–pancreas axis. Microbial transformation of primary bile acids into secondary bile acids further shapes immune responses [142,143]. These metabolites modulate dendritic cell maturation, influence macrophage polarization, and regulate the balance between proinflammatory T helper 17 cells and anti-inflammatory regulatory T cells [142,143]. Perturbations in bile acid metabolism, resulting from either microbial compositional changes or host metabolic dysfunction, can therefore propagate systemic inflammation and disturb immune homeostasis [140,144]. In humans, these relationships are primarily inferred from associative studies, and direct causal links to T1D progression remain limited.

In addition to metabolite-mediated signaling, microbial structural components may influence immune activation through host pattern-recognition pathways.

### 5.7. Pattern Recognition Receptors and Immune Activation

Recognition of microbial molecular patterns through host receptors such as toll-like receptors and nucleotide-binding oligomerization domain receptors provides another mechanistic link between dysbiosis and autoimmunity [12,24,145,146]. Persistent or inappropriate activation of these receptors leads to excessive production of proinflammatory mediators and loss of immune tolerance [146,147]. Although these pathways are mechanistically plausible and supported by experimental evidence, direct causal evidence in human T1D is limited. Moreover, altered mucosal immunoglobulin A responses observed in children predisposed to T1D suggest a breakdown in the compartmentalized regulation that normally confines microbial antigens to the gut lumen [65,148,149].

Early-life host–microbial interactions may further shape these immune pathways during critical windows of immune development.

### 5.8. Early-Life Microbiota and Immune Imprinting

Additional mechanistic evidence linking early-life microbial regulation to autoimmune diabetes has been demonstrated in non-obese diabetic (NOD) mice [150]. In this model, defective expression of the antimicrobial peptide cathelicidin-related antimicrobial peptide (CRAMP) in the neonatal colon disrupted normal microbial colonization and resulted in early-life gut microbiota dysbiosis. This alteration in microbial composition was associated with aberrant immune imprinting during a critical window of immune development. Mechanistically, the dysbiotic microbiota induced type I interferon–dependent inflammatory signaling within intestinal epithelial cells, which in turn promoted pro-inflammatory immune programming and increased susceptibility to pancreatic autoimmunity later in life [150]. Restoration of intestinal CRAMP expression, either through exogenous peptide administration or colonization with CRAMP-producing probiotic bacteria, normalized microbial community structure, suppressed aberrant interferon signaling, and reduced autoimmune diabetes incidence in NOD mice [150]. Thus, this pathway provides experimental evidence that antimicrobial peptide-dependent neonatal microbial imprinting can influence later autoimmune susceptibility. However, the extent to which CRAMP-dependent regulation of early-life microbial colonization and immune imprinting translates to human T1D remains unclear.

While multiple microbiota-dependent pathways, including SCFA production, tryptophan metabolism, bile acid transformation, and pattern recognition receptor signaling, have been implicated in the regulation of immune responses relevant to T1D, it is important to recognize that direct causal evidence in humans remains limited. Much of the current mechanistic understanding is derived from experimental models, particularly studies in non-obese diabetic mice, where targeted manipulation of microbial composition or metabolite availability has been shown to alter disease susceptibility and progression. In contrast, human studies have largely been observational and demonstrate associations between microbial alterations and disease risk rather than definitive causality. Therefore, these pathways should be interpreted as biologically plausible mechanisms supported by experimental data, but not yet conclusively established drivers of disease in humans. Future studies integrating longitudinal cohort data with mechanistic and interventional approaches will be essential to determine the extent to which these microbiota-dependent processes directly contribute to the initiation and progression of β-cell autoimmunity. Within this context, regulatory T cells may represent an important pathway through which microbial, metabolic, and immune signals may influence immune tolerance along the gut–pancreas axis.

## 6. Regulatory T Cells in the Gut–Pancreas Axis

Regulatory T cells (Tregs) constitute a central immunoregulatory population within the gut–pancreas axis and are defined as CD4^+^FOXP3^+^ T cells that enforce peripheral tolerance and constrain inappropriate immune activation [57,151,152,153]. Their suppressive function is mediated through multiple complementary mechanisms, including secretion of anti-inflammatory cytokines (such as IL-10, TGF-β, and IL-35), cytolysis via granzyme-dependent pathways, metabolic disruption through IL-2 consumption and CD39/CD73-mediated adenosine production, and direct modulation of antigen-presenting cell (APC) function via CTLA-4–dependent downregulation of co-stimulatory molecules (CD80/CD86) [154,155,156,157,158]. These coordinated processes enable Tregs to regulate both innate and adaptive immune responses across tissue compartments [156,159,160].

Tregs are developmentally and functionally heterogeneous [161,162,163,164,165]. Thymus-derived Tregs (tTregs) arise during central tolerance and are selected on the basis of intermediate-affinity recognition of self-antigens, thereby establishing a foundational layer of self-tolerance [166,167,168,169]. In contrast, peripherally induced Tregs (pTregs) differentiate from conventional CD4^+^ T cells in peripheral tissues, particularly at mucosal sites, under conditions characterized by subimmunogenic antigen presentation, TGF-β signaling, and low levels of co-stimulation [170,171,172]. The intestinal environment is especially permissive for pTreg induction, reflecting continuous exposure to dietary and microbial antigens in the presence of tolerogenic APC subsets, including CD103^+^ dendritic cells [97,173].

Within the GPA, pTregs are of particular mechanistic relevance due to their sensitivity to microbiota-derived signals [174,175,176,177]. SCFAs, especially butyrate and propionate, promote pTreg differentiation and functional stability through HDAC inhibition and G protein-coupled receptor signaling [124,178]. Within the Treg compartment, these pathways are relevant because they support FOXP3 stability and tolerogenic dendritic-cell programming [174,179]. Microbial metabolism of tryptophan generates indole derivatives that activate the AHR, a pathway that supports pTreg differentiation and interleukin-22–mediated epithelial barrier maintenance. Additional microbial and host-derived factors, including retinoic acid and bile acid metabolites, further shape Treg differentiation and function within the intestinal microenvironment.

Beyond their local effects, Tregs contribute to systemic immune regulation through migration and compartmental redistribution [180,181,182,183]. Gut-primed Tregs expressing tissue-homing receptors (such as α4β7 integrin and CCR9) can traffic to secondary lymphoid organs, including pancreatic draining lymph nodes, where they modulate antigen presentation and suppress autoreactive T-cell activation [184,185,186,187]. This trafficking provides a mechanistic link between intestinal immune conditioning and distal immune tolerance at the level of the pancreatic islet [177,188,189]. Conversely, disruption of gut-derived Treg induction or stability may reduce the availability of functionally competent Tregs in pancreatic compartments, thereby facilitating the expansion of autoreactive effector T cells [190,191,192,193].

Functional heterogeneity within the Treg compartment further influences their role in disease pathogenesis [161,194,195]. Tregs exhibit context-dependent phenotypes, including tissue-resident subsets with specialized transcriptional programs and differential sensitivity to inflammatory cues [180,182,196]. Under conditions of chronic inflammation, Tregs may undergo functional destabilization characterized by reduced FOXP3 expression, altered epigenetic regulation, and acquisition of effector-like properties, including production of pro-inflammatory cytokines such as IFN-γ or IL-17 [161,190,197]. In T1D, defects in Treg function, encompassing impaired suppressive capacity, altered signaling through IL-2 and T-cell receptor pathways, and reduced stability in inflammatory environments, have been implicated in the breakdown of immune tolerance and progression toward β-cell autoimmunity [184,185,191].

At a systems level, Tregs can operate at the intersection of microbial, metabolic, and immune networks that define the GPA [8,198,199,200,201]. Their differentiation, maintenance, and function have been associated with microbial composition and metabolite availability, epithelial barrier integrity, and the cytokine milieu [177,188,202,203,204]. Perturbations in any of these components may disrupt Treg-mediated regulation and shift the balance toward pro-inflammatory immune activation [57,205,206,207,208]. However, it is important to distinguish between experimental and human evidence. In experimental systems, particularly NOD mouse models, microbiota manipulation, microbial metabolites, and diet-derived interventions have provided causal support for microbiota-dependent modulation of Treg responses and autoimmune diabetes progression. In humans, by contrast, most studies remain observational and primarily demonstrate associations between microbiota composition, metabolic profiles, immune phenotypes, and T1D risk or progression rather than definitive causality. Accordingly, Tregs may represent a mechanistic link through which gut microbial, environmental, and host-derived factors converge on pancreatic immune regulation, but the extent to which these pathways causally drive disease susceptibility and progression in human T1D requires further longitudinal and interventional validation [12,24,174].

## 7. A Feedback Model of Microbiota–Immune Interactions

**Figure 2:**
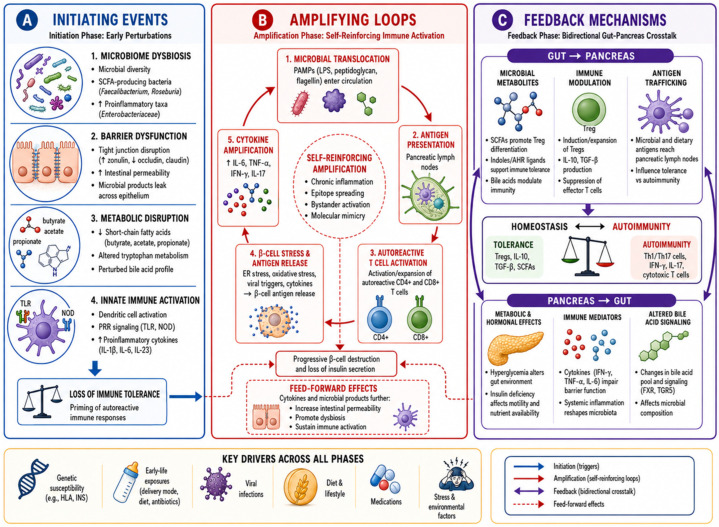
Dynamic Gut–Pancreas Axis Interactions in the Pathogenesis of Type 1 Diabetes (**A**) Initiating events: Early perturbations in gut microbial composition, epithelial barrier integrity, microbial metabolite production, and innate immune sensing may promote mucosal immune dysregulation and loss of immune tolerance. (**B**) Amplifying loops: Barrier disruption and microbial translocation may activate antigen-presenting cells, expand autoreactive CD4^+^ and CD8^+^ T cells, induce β-cell stress and antigen release, and establish cytokine-driven feed-forward inflammatory circuits that accelerate β-cell injury. (**C**) Feedback mechanisms: Reciprocal gut–pancreas signaling through microbial metabolites, antigen trafficking, cytokines, hyperglycemia, insulin deficiency, and bile acid pathways may reinforce dysbiosis and inflammation or, when regulatory pathways dominate, restore immune and metabolic homeostasis. Created in BioRender. Mittal, R. (2026) https://BioRender.com/xc2biho (accessed on 10 April 2026).

Building on the framework introduced above, the GPA operates as a feedback network in which microbial, metabolic, and immune processes interact [209]. Within this framework, microbial metabolites influence pancreatic immune regulation, while signals originating from the pancreas reciprocally shape intestinal microbial homeostasis (Figure 2). These interactions include previously described antimicrobial peptide–mediated pathways that link pancreatic signaling to microbial composition. Reciprocal signaling from the gut to the pancreas occurs through pathways involving microbial metabolites, epithelial barrier function, and immune activation, as outlined in preceding sections [209,210] (Table 1). This disruption facilitates the translocation of microbial molecules such as lipopolysaccharide, peptidoglycan fragments, and bacterial nucleic acids into the circulation [106,211].

Concurrently, inflammatory and metabolic stress originating from the pancreatic microenvironment exerts reciprocal effects on intestinal physiology [25,146]. Cytokines and reactive metabolites released during early β-cell injury alter bile acid composition, intestinal motility, epithelial turnover, and mucosal secretions [13,140]. In addition, metabolic disturbances associated with diabetes, particularly chronic hyperglycemia, can directly influence gut microbial composition and function [212,213]. Elevated glucose levels alter host–microbiome interactions and reshape the intestinal microbial ecosystem. Studies in streptozotocin-induced diabetic rat models demonstrated that hyperglycemia is associated with reduced microbial diversity and compositional shifts in gut bacterial communities, including expansion of pro-inflammatory taxa and depletion of beneficial commensal organisms involved in maintaining epithelial barrier integrity and mucosal immune regulation [212]. These findings suggest that hyperglycemia-driven dysbiosis may contribute to intestinal inflammation and metabolic dysregulation in diabetes.

Further evidence indicates that hyperglycemic metabolic environments can also induce functional remodeling of gut microbial communities [213]. Alterations in microbial metabolic pathways, including those related to carbohydrate metabolism, inflammatory signaling, and oxidative stress responses, have been observed under diabetic metabolic conditions, highlighting the impact of hyperglycemia on microbial functional activity and host immune regulation [213]. These observations indicate that metabolic disturbances arising from pancreatic dysfunction can reshape the intestinal microbial landscape and influence host immune and metabolic signaling pathways, thereby contributing to the progression of diabetes-associated complications. In turn, these microbial and metabolic alterations modify the chemical and physical conditions of the gut habitat, exerting selective pressure on microbial communities and favoring the growth of opportunistic or proinflammatory species [146]. The resulting shifts in microbial composition further amplify barrier dysfunction and systemic immune activation, creating a reinforcing feedback loop between gut and pancreas.

The transition from immune tolerance to autoimmunity can therefore be viewed as an ecological phase shift within this interconnected system [25]. Under homeostatic conditions, the host and microbiota exist in a state of mutualism in which microbial metabolites, epithelial integrity, and immune regulation are maintained in equilibrium [13,140,146]. When this balance is disturbed, either by genetic susceptibility, environmental insult, or metabolic stress, the system enters a state of dysregulated cross-talk that perpetuates inflammation and tissue destruction [25,147]. This conceptualization positions T1D as a disorder of failed host–microbial coadaptation, rather than a purely immune-mediated process [13,146]. Understanding the GPA as a self-reinforcing network offers a framework for interpreting disease heterogeneity, identifying early biomarkers of dysregulation, and developing interventions aimed at restoring ecological and immunological stability.

## 8. Therapeutic and Preventive Implications

Recognizing that alterations in the intestinal microbiota may contribute to β-cell autoimmunity in experimental systems and may also occur as a downstream consequence of disease-associated immune or metabolic changes has important implications for therapeutic innovation in T1D [12,214,215] (Table 2). This dual role positions the microbiome not merely as a biomarker of disease progression but as a modifiable target capable of influencing immune and metabolic homeostasis. Interventions that aim to reestablish equilibrium within the gut microbial ecosystem could attenuate aberrant immune activation and promote the reestablishment of peripheral tolerance [24,214,216] (Table 2).

In addition to immunomodulatory effects, emerging evidence indicates that gut microbiota can directly influence the endocrine function of pancreatic β-cells. Microbial metabolites and host–microbiome signaling pathways have been shown to modulate insulin secretion and glucose metabolism, suggesting that microbial communities contribute to the regulation of pancreatic endocrine activity. Studies investigating host–microbiome interactions demonstrated that microbial-derived signals can influence β-cell physiology and metabolic homeostasis, supporting the concept that gut microbiota participate in the regulation of endocrine pancreatic function [99]. More recent investigations further demonstrated that microbiota-targeted interventions can improve glucose regulation and modulate metabolic signaling pathways, highlighting the capacity of microbial communities to influence β-cell activity and systemic metabolic control [217]. These findings suggest that microbiota-directed therapeutic strategies may not only regulate immune responses but also support pancreatic endocrine function, highlighting the therapeutic potential of microbiome modulation in individuals with established T1D.

One promising approach involves the administration of defined probiotic or synbiotic formulations composed of commensal strains with demonstrated immunoregulatory or barrier-enhancing functions [218,219,220,221]. These microorganisms may augment SCFA synthesis, strengthen epithelial junctions, and promote regulatory immune responses. Experimental studies have provided proof-of-concept evidence that microbiota-targeted interventions may prevent autoimmune diabetes [222]. Indigenous *Clostridium* species are known to promote the differentiation of colonic regulatory T cells (Tregs), and lymphocytes primed in the intestinal environment can migrate to extraintestinal sites, including pancreatic islets, under inflammatory conditions [222]. Building on this concept, oral administration of the probiotic strain *Clostridium butyricum* CGMCC0313.1 (CB0313.1) to female NOD mice from 3 to 45 weeks of age significantly delayed diabetes onset and reduced the severity of insulitis. Immunophenotypic analyses of mesenteric lymph nodes, pancreatic lymph nodes, pancreas, and spleen revealed that CB0313.1 treatment increased Treg frequencies and restored the balance among effector T-cell subsets, including Th1, Th2, and Th17 cells, thereby promoting a less pro-inflammatory immune environment. Notably, an increased proportion of α4β7^+^ Tregs expressing the gut-homing receptor α4β7 in pancreatic lymph nodes suggested that gut-primed regulatory T cells may migrate to the pancreas and contribute to local immune regulation. In parallel, 16S rRNA gene sequencing demonstrated that probiotic treatment reshaped the gut microbiota, increasing the Firmicutes/Bacteroidetes ratio and enriching *Clostridium* clusters and other butyrate-producing bacterial taxa [222]. These findings provide experimental support for the concept that targeted probiotic modulation of the gut microbiota can influence systemic immune responses and may represent a potential strategy for preventing autoimmune diabetes.

In addition to probiotic-based approaches, dietary interventions represent another complementary strategy [223,224]. Diets rich in fermentable fibers, polyphenols, and other prebiotic substrates can enhance production of microbial metabolites, including butyrate, propionate, and acetate, with downstream effects on epithelial and immune regulation [225,226]. Beyond live microbes, increasing attention has been given to postbiotics, which are purified microbial metabolites or structural components that reproduce the beneficial effects of commensal metabolism without requiring colonization [98,227,228,229]. These agents offer advantages in terms of stability, safety, and standardization, and may allow precise control over specific metabolic pathways relevant to immune regulation.

Accumulating mechanistic evidence linking intestinal microbial communities to pancreatic immune and endocrine regulation has stimulated growing interest in translating microbiome research into clinical interventions for individuals with type 1 diabetes (T1D). Clinical studies have therefore begun to investigate microbiota-based therapeutic strategies aimed at preserving pancreatic β-cell function and modulating immune responses associated with disease progression [230,231]. Among these approaches, FMT has emerged as one of the most extensively investigated microbiome-directed interventions. In a randomized clinical trial involving individuals with recent-onset T1D, repeated FMT was associated with preservation of stimulated C-peptide secretion and stabilization of residual β-cell function during the first year following diagnosis [152]. These findings suggest that modification of intestinal microbial communities may influence disease progression and metabolic outcomes [152]. More recent clinical investigations have explored alternative microbiota-transfer strategies designed to improve safety, scalability, and patient acceptability. A pilot clinical study evaluating autologous lyophilized fecal microbiota capsules demonstrated that daily oral administration of these capsules was safe and well tolerated in individuals with T1D [214]. It was associated with stabilization of mixed-meal stimulated C-peptide levels during the intervention period, suggesting a potential role for microbiota modulation in preserving residual β-cell function [214]. These findings indicate that capsule-based microbiota delivery systems may represent a minimally invasive approach for modifying gut microbial composition and metabolic signaling pathways relevant to disease progression in T1D.

Beyond microbiota transplantation, emerging clinical and translational investigations are evaluating probiotic, prebiotic, and synbiotic interventions designed to restore microbial diversity and enhance the production of immunomodulatory microbial metabolites that regulate immune tolerance along the gut–pancreas axis [232,233,234]. Early pilot studies in individuals with T1D have suggested that modulation of gut microbial composition may influence inflammatory signaling pathways and metabolic parameters relevant to disease progression [235,236,237].

Microbiota-derived metabolites including short-chain fatty acids, bile acid derivatives, and tryptophan metabolites are increasingly recognized as key regulators of epithelial barrier integrity, immune tolerance, and host metabolic signaling pathways that influence pancreatic β-cell function. Although clinical outcomes remain heterogeneous and larger randomized trials are required to establish therapeutic efficacy, these studies collectively highlight the emerging potential of microbiota-targeted interventions as adjunctive strategies for preserving β-cell function and modifying disease progression in individuals with newly diagnosed or established T1D.

Importantly, the therapeutic impact of microbiota modulation may be dependent on the timing of intervention. Evidence from longitudinal birth cohorts suggests that the earliest stages of microbial community development coincide with immune system maturation and the establishment of mucosal tolerance. Therefore, preventive measures implemented during infancy or early childhood, before or shortly after the appearance of islet autoantibodies, may yield the highest efficacy. Maternal microbial modulation during pregnancy is also under investigation as a means of influencing the neonatal microbiome and shaping immune imprinting.

A precision medicine framework will be essential to translate these insights into individualized therapies. Integrating multi-omic analyses encompassing microbial composition, metabolomic profiles, and host immune phenotypes could enable the identification of patient subgroups that are most likely to benefit from specific microbiota-directed interventions. Machine learning-based approaches may further refine predictive models by linking microbiome signatures to clinical outcomes, facilitating personalized preventive or therapeutic programs [238].

Future therapeutic paradigms may also involve combination strategies in which microbial modulation is paired with immune tolerance induction. For example, antigen-specific immunotherapies designed to desensitize autoreactive lymphocytes might achieve greater success when delivered alongside interventions that restore intestinal immune homeostasis. This synergistic approach could enhance regulatory network stability and protect residual beta cell function.

Despite these prospects, significant challenges remain. The human microbiome exhibits substantial interindividual variability influenced by genetics, geography, diet, and medication use, which complicates the reproducibility of microbial interventions. The long-term ecological consequences of altering the microbiota are not fully understood, and there are unresolved regulatory and ethical considerations surrounding the use of live biotherapeutic products in pediatric populations. Moreover, many existing clinical trials are limited by small sample sizes, heterogeneous study designs, and a lack of standardized endpoints.

To overcome these limitations, rigorously controlled, multicenter studies incorporating mechanistic endpoints are needed to clarify causal relationships and optimize intervention protocols. Such efforts should include longitudinal follow up to determine the durability of microbial and immunologic changes. Establishing clear safety parameters and robust regulatory frameworks will be essential to ensure the responsible development of microbiota-based therapies. Ultimately, a comprehensive understanding of the gut microbial ecosystem and its communication with the pancreas will provide the foundation for novel preventive and therapeutic strategies aimed at halting or reversing the autoimmune cascade that underlies T1D.

## 9. Concluding Remarks and Future Directions

The evolving understanding of the GPA necessitates a fundamental reconsideration of causality in the pathogenesis of T1D. Accumulating data suggests that the intestinal microbiota is not merely a secondary consequence of metabolic dysfunction or immune activation. Rather, experimental systems support an active role for microbial pathways in shaping the initiation, propagation, and chronicity of β-cell autoimmunity, whereas human studies indicate a potential contributory role that remains to be confirmed causally [86,146,214]. The host microbial interface represents a highly dynamic ecosystem in which microbial metabolites, immune mediators, and epithelial barrier functions are interwoven into a complex regulatory network [147,239]. Perturbations within this system may tip the balance from immune tolerance to sustained inflammation, ultimately resulting in irreversible β-cell destruction [25].

Future research should aim to elucidate the precise temporal and mechanistic relationships between microbial alterations and autoimmune processes. Longitudinal birth cohorts that integrate multi-omics profiling including metagenomics, transcriptomics, metabolomics, and immunophenotyping will be crucial to identify microbial signatures predictive of disease onset and progression. Experimental models capable of reproducing early-life microbial perturbations are equally important for testing causal hypotheses and for dissecting the molecular pathways linking the gut environment to pancreatic immune activation. Such studies should also account for host variables including genetic susceptibility, diet, antibiotic exposure, and viral infections which collectively influence microbial assembly and immune maturation.

Advances in computational modeling and systems biology now make it possible to construct integrative frameworks that capture the dynamic interactions among microbial communities, host metabolism, and immune networks [96,240,241]. These models can help to define the thresholds at which ecological disturbances become pathophysiologically relevant and may guide the design of microbiota-targeted interventions [96,242,243,244,245]. Translational studies combining microbial modulation with immunotherapeutic approaches may help restore regulatory host–microbial interactions within the GPA. Early life interventions aimed at promoting microbial diversity and functional resilience could become a cornerstone for primary prevention, while tailored postbiotic or consortia-based therapies may complement immune tolerance induction in individuals at advanced stages of autoimmunity.

Ultimately, adopting an ecological perspective on T1D reframes the disease as a breakdown of symbiosis between the host and its microbial partners. By restoring the equilibrium of this complex axis, it may be possible to attenuate immune dysregulation and preserve residual β-cell function. The integration of microbial science into immunology, endocrinology, and metabolic medicine will be essential to translate these conceptual advances into practical strategies for the prevention and long-term management of T1D.

Future progress will depend on resolving key uncertainties related to causality, temporal dynamics, and therapeutic tractability within the gut–pancreas axis. A central priority is to determine whether specific microbial configurations and metabolite profiles directly influence β-cell–directed autoimmunity in humans, and to define the developmental windows during which such effects are most consequential. Addressing these questions will require integrative study designs that combine longitudinal human cohorts with mechanistic and interventional approaches. In parallel, the identification of reproducible microbial and host-derived biomarkers that enable risk stratification and therapeutic monitoring remains an important unmet need. Standardization of microbiome-targeted interventions, along with rigorous evaluation in controlled clinical settings, will be essential to establish their safety, efficacy, and durability. Advances in multi-omic integration and system-level modeling are expected to further enable the linkage of microbial dynamics with patient-specific immune trajectories, thereby supporting the development of precise, mechanism-based strategies for the prevention and clinical intervention of T1D.

## Figures and Tables

**Table 1:** Microbiota-Driven Mechanisms implicated in the Gut–Pancreas Axis in Type 1 Diabetes.

Potential Mechanistic Pathway	Key Findings	Evidence Source	Level of Support	References
SCFA-mediated immune regulation	Produced by commensal bacteria such as *Faecalibacterium* and *Roseburia* Promote regulatory T-cell differentiation Enhance epithelial barrier integrity Suppress inflammatory responses Reduced SCFA availability is associated with impaired immune tolerance and enhanced β-cell–directed autoimmunity	Human + Animal	Human: Associative; Animal: Experimental/Interventional	[15,29,74]
Intestinal barrier dysfunction	Increased intestinal permeability Altered tight junction integrity Reduced mucin and antimicrobial peptide expression Enhanced antigen translocation Associated with systemic immune activation linked to β-cell autoimmunity	Human + Animal	Human: Associative; Animal: Experimental	[29,74]
Pattern recognition receptor activation	Activation of toll-like and NOD-like receptor pathways Induction of inflammatory signaling and cytokine production Enhanced antigen presentation Contributes to proinflammatory responses associated with β-cell destruction	Animal + Limited Human	Experimental	[19,29]
Molecular mimicry	Microbial peptide homology with β-cell antigens Activation of cross-reactive autoreactive T cells Breakdown of immune tolerance Contributes to initiation and amplification of autoimmunity	Animal	Experimental	[19,97]
Tryptophan metabolism and AHR signaling	Microbial metabolites regulate IL-22 production Modulate mucosal immune balance via AHR activation Support epithelial barrier function Disruption associated with immune dysregulation and increased susceptibility to autoimmunity	Human + Animal	Human: Associative; Animal: Experimental	[74,98]
Bile acid metabolism	Microbial transformation of bile acids Influences immune cell differentiation Regulates inflammatory balance Dysregulation contributes to immune dysfunction	Human + Animal	Human: Associative; Animal: Experimental	[74]
Antimicrobial peptide regulation	Microbiota-dependent regulation of antimicrobial peptides such as CRAMP Influences microbial composition Supports immune tolerance and homeostasis Impairment associated with dysbiosis and increased disease susceptibility	Animal	Experimental	[19,99]
Microbial translocation and antigen presentation	Translocation of microbial components across compromised intestinal barrier Activation of antigen-presenting cells Promotion of pro-inflammatory signaling Cross-presentation of β-cell antigens linking gut-derived signals to autoimmunity	Animal > Human	Experimental (strong in animal models; limited human data)	[19]

Abbreviations: SCFA: short-chain fatty acid; AHR: aryl hydrocarbon receptor; CRAMP: cathelicidin-related antimicrobial peptide; NOD: nucleotide-binding oligomerization domain; IL: interleukin.

**Table 2:** Microbiota-Targeted Therapeutic Strategies in Type 1 Diabetes.

Intervention	Proposed Mechanism	Evidence Type	Stage of Development	Key Limitations/Considerations
Probiotics	Modulate microbial composition and metabolic activity Enhance SCFA production and regulatory T-cell responses Improve epithelial barrier integrity May reduce pro-inflammatory signaling	Animal + Early Human	Preclinical → Early Clinical	Strain-specific effects Variable reproducibility in humans Limited large-scale clinical trials
Prebiotics and dietary fiber	Promote growth of beneficial commensal bacteria Increase endogenous SCFA production Support epithelial barrier function and immune balance Influence microbial metabolic pathways	Human + Animal	Early Clinical	High inter-individual variability Diet adherence-dependent effects Variable response across populations
Postbiotics	Deliver microbial-derived metabolites or bioactive compounds Directly modulate immune and epithelial responses Avoid risks associated with live microorganisms	Preclinical	Preclinical	Limited clinical validation Uncertain pharmacokinetics and dosing Mechanistic pathways not fully characterized
Fecal microbiota transplantation	Restructures microbial ecosystem and restores diversity Alters microbial metabolic and immune signaling pathways Potential to shift dysbiotic communities toward a balanced state	Human + Animal	Early Clinical	Donor variability Regulatory and standardization challenges Long-term safety remains uncertain
Synbiotics	Combine probiotic and prebiotic effects Enhance microbial colonization and metabolic activity Promote synergistic modulation of host–microbiome interactions	Limited Human + Animal	Early Clinical	Limited controlled clinical studies Variability in formulations Inconsistent outcomes across studies
Microbiome-based precision approaches	Personalized modulation based on individual microbiome and host characteristics Potential to optimize targeted therapeutic responses Integrates multi-omics and host–microbiome interactions	Conceptual + Early Research	Preclinical	Requires extensive validation High complexity and cost Limited current clinical applicability

## Data Availability

No new data were created or analyzed in this study.

## Abbreviations

The following abbreviations are used in this manuscript:

AHR	aryl hydrocarbon receptor
AMP	antimicrobial peptide
CRAMP	cathelicidin-related antimicrobial peptide
CVB	coxsackievirus group B
FMT	fecal microbiota transplantation
FXR	farnesoid X receptor
GAD65	glutamic acid decarboxylase 65
GPA	gut–pancreas axis
GPR	G-protein-coupled receptor
HDAC	histone deacetylase
HLA	human leukocyte antigen
IA-2	islet antigen-2
IL	interleukin
LPS	lipopolysaccharide
MAMP	microbe-associated molecular pattern
MASLD	metabolic dysfunction-associated steatotic liver disease
MUC	mucin
NOD mice	non-obese diabetic mice
NOD2	nucleotide-binding oligomerization domain-containing protein 2
pTreg	peripherally induced regulatory T cell
SCFA	short-chain fatty acid
T1D	type 1 diabetes
TGR5	Takeda G-protein-coupled receptor 5
TLR	Toll-like receptor
Treg	regulatory T cell (used as the standard collective abbreviation throughout)
tTreg	thymically derived regulatory T cell
ZO-1	zonula occludens-1
